# Contribution of Cytochrome P450 and *ABCB1* Genetic Variability on Methadone Pharmacokinetics, Dose Requirements, and Response

**DOI:** 10.1371/journal.pone.0019527

**Published:** 2011-05-12

**Authors:** Francina Fonseca, Rafael de la Torre, Laura Díaz, Antonio Pastor, Elisabet Cuyàs, Nieves Pizarro, Olha Khymenets, Magí Farré, Marta Torrens

**Affiliations:** 1 Institut de Neuropsiquiatria i Addiccions - Parc de Salut Mar, Barcelona, Spain; 2 Universitat Pompeu Fabra (CEXS-UPF), Barcelona, Spain; 3 Disorders by Use of Substances Research Group, Neuropsychopharmacology Research Program, Institut Municipal d'Investigació Mèdica (IMIM-Hospital del Mar Research Institute), Barcelona, Spain; 4 Human Pharmacology and Clinical Neurosciences Research Group, Neuropsychopharmacology Research Program, Institut Municipal d'Investigació Mèdica (IMIM-Hospital del Mar Research Institute), Barcelona, Spain; 5 CIBER de Fisiopatología de la Obesidad y Nutrición (CB06/03), Hospital Clínico Universitario Santiago de Compostela, Santiago de Compostela, Spain; 6 Pharmacology Department, Autonomous University of Barcelona, Barcelona, Spain; 7 Psychiatric Department, Autonomous University of Barcelona, Barcelona, Spain; Dr. Margarete Fischer-Bosch Institute of Clinical Pharmacology, Germany

## Abstract

Although the efficacy of methadone maintenance treatment (MMT) in opioid dependence disorder has been well established, the influence of methadone pharmacokinetics in dose requirement and clinical outcome remains controversial. The aim of this study is to analyze methadone dosage in responder and nonresponder patients considering pharmacogenetic and pharmacokinetic factors that may contribute to dosage adequacy. Opioid dependence patients (meeting Diagnostic and Statistical Manual of Mental Disorders, [4^th^ Edition] criteria) from a MMT community program were recruited. Patients were clinically assessed and blood samples were obtained to determine plasma concentrations of *(R,S)-*, *(R)* and *(S)-* methadone and to study allelic variants of genes encoding CYP3A5, CYP2D6, CYP2B6, CYP2C9, CYP2C19, and P-glycoprotein. Responders and nonresponders were defined by illicit opioid consumption detected in random urinalysis. The final sample consisted in 105 opioid dependent patients of Caucasian origin. Responder patients received higher doses of methadone and have been included into treatment for a longer period. No differences were found in terms of genotype frequencies between groups. Only *CYP2D6* metabolizing phenotype differences were found in outcome status, methadone dose requirements, and plasma concentrations, being higher in the ultrarapid metabolizers. No other differences were found between phenotype and responder status, methadone dose requirements, neither in methadone plasma concentrations. Pharmacokinetic factors could explain some but not all differences in MMT outcome and methadone dose requirements.

## Introduction

Maintenance treatment of opioid dependence with methadone is a well known pharmacotherapy approach. However, there is a large interindividual variability in clinical outcomes among subjects in methadone maintenance treatment (MMT) [1]. In fact, between the 30% and 80% of patients receiving methadone are poor responders when retention in the MMT and/or illicit opioid use are considered as the main outcome variables [2], [3]. Several factors like, poor coping self-efficacy [4], mood states [5], genetic polymorphisms in drug metabolizing enzymes [6], and methadone pharmacokinetics [5] have been suggested as contributing factors. One of the main factors related to MMT success is the dose of methadone provided [7]–[9].

Although a strong correlation between methadone dose and concentrations in plasma has been reported [10], this relationship may not be linear, and it has been shown that the determination of methadone plasma concentrations and their enantiomers is not useful to predict illicit opioid use, nor opioid withdrawal symptoms [11], [12]. Although methadone is usually administered as a racemate (a 50∶50 mixture of two enantiomers, *(R)*- and *(S)*-methadone), the *(R)*-enantiomer accounts for the majority of opioid agonist effects [13]. The metabolic disposition of methadone also displays a certain degree of enantioselectivity.

Methadone is metabolized by the cytochrome P450 system being major contributing isoenzymes CYP3A4, CYP2B6 and to a lesser extent CYP2D6 [14]. Other isoenzymes, such as CYP1A2, CYP2C8, CYP2C9 and CYP2C19 could also be involved in methadone metabolism but there are contradictory data [15]. The involvement of different isoenzymes of cytochrome P450 in methadone metabolism should be considered to understand the clinical pharmacology of this substance.

### CYP3A 4/5

CYP3A4 is the major isoenzyme of cytochrome P450 involved in EDDP (2-ethylidene-1,5-dimethyl-3,3-diphenylpyrrolidine) formation from methadone [16], [17] in a non-enantioselective manner [16], [18], [19]. Induction of CYP3A4 at the beginning of MMT probably explains, at least in part, the increased EDDP/methadone ratio [20] and justifies the need of dosage adaptation. The activity of this enzyme is highly variable among individuals, and can be affected by environmental and genetic factors. The most studied allelic variant is the CYP3A4*1B allele, which was associated with a 1.5-fold increase in transcription in vitro. In an in vivo study by Crettol et al. [14] the carriers of the CYP3A4*1B variant presented a 1.4-fold increase for *(S)-*methadone and 1.1-fold increase for *(R)*-methadone; also, the CYP3A4*1B variant carriers have more probability to be in the low-dose group, suggesting that they have higher methadone plasma concentrations and require lower methadone doses.

The hepatic expression of CYP3A5 is bimodally distributed, indicating the existence of genetic polymorphisms [21]. Several genetic variants have been described for CYP3A5, and the most common, the CYP3A5*3 allele, causes the loss of CYP3A5 activity. Thus, only individuals carrying at least one CYP3A5*1 allele express large amounts of CYP3A5 [22], [23]. This polymorphism has been reported to influence total CYP3A activity and shows ethnic differences in its frequency [24]. Thus, a substantial change in CYP3A5 activity might influence the pharmacokinetics of CYP3A substrates [25]. In fact, it has been shown that patients with CYP3A5*1/*1 and *1/*3 genotypes require a significantly higher sirolimus daily dose to achieve the same blood concentration at steady-state as *3/*3 patients [26]. Furthermore, in most cases, subjects expressing CYP3A5 also express very high levels of CYP3A4. Therefore, even if CYP3A5 was not shown to play an active role in methadone metabolism in vitro [27], [28] since it may represent up to 50% of the total hepatic CYP3A content in subjects expressing it [23], and in view of the fact that subjects expressing it also have very high levels of CYP3A4 activity, it might be an important contributor to the interindividual variability in methadone metabolism.

### CYP2D6

CYP2D6 is expressed in the liver and is subject to genetic polymorphism. In vitro studies show a minor role of CYP2D6 in the formation of EDDP from methadone [16] with a enantioselectivity towards *(R)-*methadone [29]. However, observed pharmacokinetic interactions between methadone and CYP2D6 inhibitors seem to indicate a more relevant contribution to methadone metabolic disposition [30], [31]. CYP2D6 displays a genetic polymorphism with as many as one hundred allelic variants. Among them, variants *3 to *8 are nonfunctional, *9, *10, *41 have reduced functionality, and *1, *2, *35, *4 and *41 can be duplicated, resulting in an increased expression of functional (or non-functional) CYP2D6 protein. Allele combinations determine CYP2D6 phenotype, which includes the poor metabolizer (PM; two non functional alleles), the extensive metabolizer (EM; at least one functional allele), the intermediate metabolizers (IM; two decreased activity alleles) and the ultra-rapid metabolizer (UM; multiple copies of a functional allele and/or allele with promoter mutation). These phenotypes have been related with methadone plasma concentrations [32], [33]. Clinical studies showed an influence of CYP2D6 phenotype in lower trough *(R,S)-*methadone plasma concentrations [14] and in the reported satisfaction with methadone treatment [6]. Discrepancies between genotype and in vivo CYP2D6 activity in MMT patients have been described [34]; the authors postulated that the finding was consistent with inhibition of CYP2D6 activity by methadone [35].

### CYP2B6

CYP2B6 shows a cross-regulation with CYP3A4, UGT1A1 and several hepatic drug transporters by the nuclear receptors pregnane X receptor (PXR) and constitutive androstane receptor (CAR). This is of relevance since CYP3A4 and drug transporters are involved in methadone metabolic disposition [36].

In vitro and in vivo studies have shown that CYP2B6 is a contributor to methadone metabolism [15] with an observed enantioselectivity towards de *(S)-*enantiomer [18], [19], [37]. In vivo studies also demonstrated that CYP2B6 genotype influences methadone plasma concentrations, mainly *(S)*-methadone. Multiple SNPs within the CYP2B6 gene, located on chromosome 19q13.2, have been described [14], [38]. The CYP2B6 genotype *6/*6 is associated with a decreased activity of the protein *in vitro* [36] and in previous studies of patients in methadone treatment has been related with high *(S)-*methadone plasma concentrations, with no significant effects in *(R)-*methadone plasma concentrations [14], [37]. Genotype differences were not associated with MMT response, nor methadone dose requirements. The stereoselectivity towards the non active enantiomer could explain these results [14].

### Other cytochromes

Available *in vivo* and *in vitro* data suggest that CYP1A2 is not involved in methadone metabolism [15]. Other enzymes have been recently evaluated in relationship with methadone metabolism: CYP2C19 and CYP2C9. Whereas some authors describe an influence in methadone metabolic disposition [18], [39], other authors haven't found an influence on enantiomer methadone plasma concentrations [14], [37].

### P-glycoprotein

Methadone is a substrate of P-glycoprotein (P-gp) which shows a weak stereoselectivity towards the *(S)-*enantiomer [40]. It is a trans-membrane protein of 1280 amino acids. It is expressed in tissues with a barrier function [41]. The activity of P-gp in intestines and the brain blood barrier has been shown to be of some relevance in determining methadone concentrations [15]. P-gp is encoded by the multidrug resistance 1 (*ABCB1*) gene on chromosome 7p21. This gene is highly polymorphic and a number of variants have been associated with drug response [42]. The majority of studies focused on a non-synonymous SNP in exon 26, 3435C>T; the homozygosity to the allele T showed lower in vivo duodenal P-gp expression [42]; also, the *ABCB1* 3435T allele may alter the stability of *ABCB1* mRNA and is associated with lower mRNA concentrations [43], [44].

Genetic variability of *ABCB1* and effects on MMT have already been studied. One study [45] with 60 opioid dependent subjects in MMT showed that *ABCB1* genetic variability influenced daily methadone requirements. Other authors [14], [46] showed an influence in *(R,S)-*methadone plasma concentrations, but they didn't found any influence in therapeutic response.

Several studies have been conducted to assess the influence of haplotypes on the clinical response to methadone, with contradictory findings, probably related to methodological differences (methadone formulations, treatment duration and previous exposure to methadone) [15].

The study of patients' genetic polymorphisms in genes encoding for methadone-metabolizing enzymes and transporters has been an active area of research but the clinical relevance in MMT outcome is still unclear [15], [47]. A cross-sectional study was designed to assess the influence of *ABCB1* and cytochrome P450 genetic variability on methadone pharmacokinetics, dose requirements, and clinical response in opioid dependent patients included in a MMT program.

## Methods

### Ethics Statement

Written informed consent was obtained from each subject after they had received a complete description of the study and been given the chance to discuss any questions or issues. The study was approved by the Ethical and Clinical Research Committee of our institution (CEIC-IMAS).

### Design and Patients

The study recruited opioid dependence patients who met criteria for opioid dependence following the Diagnostic and Statistical Manual of Mental Disorders (4th Edition) [DSM-IV] from a MMT program (MMT community program, CAS Barceloneta, Barcelona, Spain). The main characteristics of the MMT provided included: clinical management with individual counseling to encourage drug abstinence, methadone dosages as required (no restrictions for upper limit) and no restriction on treatment duration. Forced discharge occurred only as a result of patients' violent behavior or drug trafficking.

To be eligible for the study, patients had to be Caucasian, enrolled in MMT for at least four months, and receiving a stable methadone dose for the last two months. Exclusion criteria were as follows: language-related barriers, severe cognitive impairment, and any medical condition that would interfere with research assessments and refusal to take part in the study.

### Clinical Assessment

A close-ended questionnaire was used to record patients' socio-demographic characteristics, serological status (Human Immunodeficiency Virus [HIV], Hepatitis C Virus [HCV]), history of substance use, and previous psychiatric pharmacological treatment as well as other concomitant treatments. Substance use disorders and other psychiatric disorders were diagnosed according to DSM-IV criteria, using the Spanish version of the Psychiatric Research Interview for Substance and Mental Disorders (PRISM-IV) for axis I and II (borderline and antisocial personality disorders) [48], [49]. The degree of addiction-related impairment was assessed using the Spanish version of the Addiction Severity Index (ASI) [50], [51].

The use of illegal opiates was evaluated retrospectively by reviewing the results of the last 4 urine tests performed over 2 months before study inclusion. Urinalyses for the detection of heroin consumption were carried out at the centre, 1 day at random every 1 or 2 weeks, under supervision of the nursing staff. It was considered that illegal opiates had been used when 2 or more urinalyses tested positive for morphine metabolites (in the last 4 drug tests in urine). Determination of morphine and codeine metabolites in urine was performed by a gas chromatography—mass spectrometry method [52]. This method allows the identification of 6-monoacetylmorphine in urine, which can be used as a confirmatory marker of heroine abuse. These results were used to group patients as responders (all drug tests were negative) and nonresponders (2 or more positive drug tests). Because the definition of the Responder and Nonresponder phenotype is difficult to establish, it was decided to exclude subjects with subthreshold urine controls, that is, only one positive urine test in the last four screening procedures.

### Plasma Samples Analysis

A blood sample (5 mL) was taken 24 hours after the last supervised methadone oral administration. Due to the differences in the opioid effect and metabolism between methadone enantiomers, we decided to analyze plasma concentrations for both enantiomers separately and for total methadone plasma concentrations. The *(R)-*, *(S)-* and *(R,S)-* methadone plasma concentrations were determined by capillary electrophoresis technique (CE-UV) after a liquid-liquid extraction of samples with tert-butylmethylether. A capillary electrophoretic system (CE, ^3D^Hewlett-Packard) equipped with a diode-array detector (UV) was used for the enantioselective determination of methadone. *(S)-*dextrorphan and *(R)-*levorphanol were used as internal standards (I.S.) for (*S*)- and (*R*)-methadone, respectively. After a liquid-liquid extraction of 1 mL of plasma with *tert-butylmethylether* [12], resolution of the enantiomers was performed in an untreated fused-silica capillary of 48.5-cm total length (40-cm effective length) and a standard 50-µm optical path length cell. A constant voltage of 25 kV was applied and the cartridge temperature was maintained at 16°C, The diode-array detector was set to monitor the signal at 204 nm. Resolution was performed by using 1 mM heptakis-(2,6-di-O-methyl)-β-cyclodextrin in 100 mM H_3_PO_4_, pH = 2.5 as a running buffer [53].

Calibration curves were prepared for each analytical batch with appropriate volumes of the corresponding racemic mixture working solutions added to test tubes containing 1 mL of drug-free plasma, and were linear over 100–500 ng/mL concentration range of the corresponding enantiomers. Control plasma samples containing 150 (low control) and 350 ng/mL (high control) of methadone enantiomers were prepared in drug-free plasma and were kept frozen at −20°C in 1 mL aliquots until their use.

Peak-area ratios between compounds and I.S. were used for calculations. A weighted (1/concentration) least-square regression analysis was used (SPSS for Windows, version 14.0). Extraction efficiencies for (*R*)-methadone and *(S)-*methadone were calculated by comparing the peak areas of equal concentrations of drug extracted and non-extracted, being 99.8 and 86.7%, respectively. Four replicate analyses were performed with plasma samples corresponding to the first level of concentrations of the calibration curves, and 3 and 10 standard deviations (SD) of the calculated concentrations at this calibration level were used for estimating the limits of detection (LOD) and quantification (LOQ), respectively, being LOD 25.9 and 23.2 ng/mL and LOQ 78.5 and 70.2 ng/mL for (*R*) and (*S*)-enantiomer, respectively. Precision was calculated as the relative standard deviation (RSD) of the quality control samples concentrations and there were 8.9% and 10.2% for (*R*)- and (*S*)-methadone, respectively. Accuracy is expressed as the relative error of the calculated concentrations, being 8.2 and 10.9% for (*R*) and (*S*)-methadone, respectively.

### Genetic Analysis

A collection of 20 mL of blood was done to extract DNA from leukocytes to evaluate allelic variants of genes encoding the following proteins: cytochrome P450 3A5 (*CYP3A5*); cytochrome P450 2D6 (*CYP2D6*); cytochrome P450 2B6 (*CYP2B6*), cytochrome P450 CYP2C9 (*CYP2C9*), cytochrome P450 CYP2C19 (*CYP2C19*), and the Multidrug Resistance 1 transporter (*ABCB1*) [42]. The genotyping of all mentioned genes but CYP2B6 was performed using a DNA microarray (Progenika Biopharma, Derio, Spain). Details on the allelic variants monitored per gene as well as performance of the microarray have been previously described [54]. Briefly, target DNA for hybridization was prepared by amplification of all genes except CYP2D6 in several multiplex PCR reactions. The gene CYP2D6 was amplified together with a shorter deletion-specific fragment in a long-range PCR reaction. Similarly, a separate long-range multiplex PCR reaction with the CYP2D6 gene and a short duplication-specific fragment was carried out for the identification of individuals carrying multiple copies of the CYP2D6 gene.

CYP2B6 genotyping of two SNP positions was performed by TaqMan 5′-nuclease chain reaction assay using commercially available kit for 516G→T (TaqMan Drug Metabolism Genotyping Assay, Applied Biosystems, Foster City, CA) and previously published probes (MGB TaqMan Probes®, Applied Biosystems) and primers for 785A→G [55]. The PCR reaction was performed according to the manufacturer instructions on ABI PRISM 7900 sequence detection system (Applied Biosystems). The genotype for CYP2B6 was defined by haplotype combining both tested SNPs according to the earlier published determination [56]. Hence, homozygous genotypes *1/*1, *4/*4 and *6/*6 correspond to the haplotypes defined by combination of 516GG with 785AA, 516GG with 785GG, and 516TT with 785GG, respectively. Correspondingly, the combination of SNPs for heterozygous genotypes were 516GG with 785AG for *1/*4 and 516GT with 785 GG for *4/*6. For combination of 516GT with 785AG detected in 51 participant of this study, there were two possible genotypes, *1/*6 and *4/*9. Alleles *9 and *4 have of very low frequency among Spaniards (≤1.4% and ≤6.2% respectively) [57] and all carriers of these combined alleles were assigned as *1/*6 heterozygous.

The genotype distribution and corresponding allelic variants frequency were calculated.

### Statistical Analysis

Descriptive statistics of all variables of interest are presented as means and standard deviation (SD) in case of quantitative variables, and by absolute and relative frequencies in case of categorical variables. Differences in sociodemographic and clinical characteristics between groups were examined using Chi-square, One-Way ANOVA and T student (when appropriate) tests. Differences in genotype and phenotype frequencies among responders and nonresponders were assessed by Chi-square test. The phenotypes were compared with respect to methadone dose and plasma concentrations using one-way ANOVA together with Tukey post hoc analysis for pairwise comparisons. All analyses were performed with the statistical software package SPSS (SPSS Inc., Chicago, IL), version 14.0.

## Results

### Clinical Characteristics of Patients

From 169 eligible patients, 12 were non Caucasian and were excluded. Reliable information on patients' medical history and on the use of concurrent medication was obtained from 105 patients (71% male; mean age 38 years [SD = 8]) by personal interview and by review of the clinical records. The characteristics of patients (already split in responders and nonresponders) are represented in **Table 1**. The mean methadone dose of patients included in the study was 98 mg/day (SD = 64). All but 2 patients were smokers and 65% were taking concomitant treatments. Responders and nonresponders showed similar characteristics except of, days of heroin use in the last 30 day (responders 0 days [SD = 1] vs. nonresponders 16 days [SD = 10]), methadone dosage (responders 109 mg/day [SD = 68] vs. nonresponders 72 mg/day [SD = 43], p = 0.007); The lower dose of nonresponder patients cannot be explained by restrictions for upper limit in methadone dosage in the framework of the MMT. Also, patients groups showed differences in terms of months in methadone (Responders 52 months [SD = 49] vs. nonresponders 21 months [SD = 32], p = 0.001), and in the Drug Use ASI scale and Legal Problems ASI scale.

**Table 1:** Main sociodemographical and clinical characteristics of responder and nonresponder patients groups.

	Responders	Nonresponders	P^a^
	N = 76	N = 29	
Male (%)	53 (70)	21 (72)	1.000
Age, mean ± SD	39±7	36±9	0.076
Years at school ± SD	9±3	8±3	0.060
Single (%)	30 (41)	13 (45)	0.629
Criminal background (%)	40 (54)	18 (62)	0.248
Live with family (%)	58 (78)	19 (66)	0.764
Employed (%)	22 (30)	10 (42)	0.205
HIV+(%)	31 (41)	9 (31)	0.380
HCV+(%)	59 (78)	18 (62)	0.139
Lifetime psychiatric comorbidity (%)	45 (74)	14 (48)	0.416
Months of heroin use ± SD	144±80	121±67	0.192
Days of heroin 30 days ± SD	0±1	16±10	**<0.001**
Days of cocaine 30 days ± SD	2±6	7±12	0.123
Nicotine cigarettes/day ± SD	22±11	26±13	0.172
Concomitant medication (%)			
benzodiazepines	39 (51)	9 (31)	0.080
antiretrovirals	13 (17)	5 (17)	1.000
anticonvulsants	9 (12)	0 (0)	0.060
SSRI	13 (17)	1 (3)	0.106
other antidepressant (non-SSRI)	9 (12)	4 (14)	0.750
antipsychotics	14 (18)	3 (10)	0.388
antibiotics	6 (8)	1 (3)	0.670
any concomitant medication	53 (70)	15 (52)	0.110
Months in methadone ± SD	52±49	21±32	**0.001**
Methadone dosage (mg/day) ± SD	109±68	72±43	**0.007**
Methadone plasma concentrations (ng/ml) ± SD^b^			
Total *(R,S)-*methadone	587±501	443±246	0.121
*(R)-*methadone	311±259	238±131	0.136
*(S)-*methadone	276±288	205±121	0.370
ASI scores ± SD			
General Health	3±2	4±2	0.184
Work	4±3	3±3	0.670
Alcohol Use	1±2	1±1	0.127
Drug Use	4±2	6±2	**0.001**
Legal	1±2	3±3	**0.001**
Social	3±3	3±2	0.789
Psychological	3±3	3±3	0.855

a Bold numbers indicate statistically significant differences between patients.

b Plasma concentrations of methadone were obtained from 79 subjects (65 responders and 14 non-responders).

SD = standard deviation; HIV = human immundeficiency virus; HCV = hepatitis C virus; SSRI = Selective Serotonin Reuptake Inhibitors; ASI = Addiction Serverity Index.

Plasma samples were obtained from 79 patients. There were no differences between these 79 patients from which we obtained plasma and the rest (n = 26, 11 responders and 14 nonresponders) of patients included in the study samples in terms of sociodemographic, neither medical nor psychopathological characteristics. Blood samples for genotyping were usually obtained at the inclusion of patients in the MMT. Blood samples for methadone determination were obtained once the patient was enrolled for 4 months at the MMT and dose was stabilized (according to inclusion criteria) in the MMT for 2 months. The main reason for not obtaining blood samples from all patients once dose was stabilized is the lack of cooperation for sample withdrawal. Although it did not reach statistical significance, Responder patients presented higher methadone plasma concentrations of both, (*R*)- (responders 311 ng/ml [SD = 259] vs. nonresponders 238 ng/ml [SD = 131], p = 0.136) and (*S*)-methadone (responders 276 ng/ml [SD = 268] vs. nonresponders 205 ng/ml [SD = 121], p = 0.370) and, also, (*R,S*)-methadone (responders 587 ng/ml [SD = 501] vs. nonresponders 443 ng/ml [SD = 246], p = 0.121). Globally this trend reflects differences of about 25% of methadone dose between responder and nonresponder patients.

### Genotypes and Phenotypes

The frequencies of genotypes and allelic variants screened for are represented in **Table 2** and those of the different phenotypes are represented in **Table 3**. No differences were observed in the distribution of genotypes and phenotypes for genes evaluated among responders and nonresponders patients except for an overrepresentation of UM subjects of *CYP2D6* in responder patients. (7% in responders versus 0% in nonresponder patients; p = 0.032). When the *(R,S)-*methadone plasma concentrations were divided by the daily methadone dose provided, no differences were found in terms of genotypes nor phenotypes. Regarding to CYP2B6*6, in our sample, 5 patients were *6 homozygous carriers; 4 of them correspond to responder patients, and only one was classified as nonresponder.

**Table 2:** Genotype frequencies of *CYP3A5, CYP2D6, CYP2B6, CYP2C9, CYP2C19 and ABCB1* between responder and nonresponder groups.

	Responders	Nonresponders	P
	N = 76 (%)^a^	N = 29 (%)^a^	
*CYP3A5* Genotype			0.446
*1/*1	1 (1)	1 (3)	
*1/*3	11 (15)	2 (7)	
*3/*3	64 (84)	26 (90)	
*CYP2D6* Genotype			0.211
*1/*1	4 (5)	2 (7)	
*1/*2	12 (16)	4 (14)	
*1/*3	2 (3)	0 (-)	
*1/*4	16 (21)	3 (10)	
*1/*5	1 (1)	0 (-)	
*1/*6	1 (1)	0 (-)	
*1/*9	2 (3)	0 (-)	
*1/*10	2 (3)	0 (-)	
*1/*41	1 (1)	3 (10)	
*2/*2	6 (8)	2 (7)	
*2/*3	1 (1)	0 (-)	
*2/*4	9 (12)	6 (21)	
*2/*5	1 (1)	1 (3)	
*2/*6	1 (1)	1 (3)	
*2/*9	2 (3)	0 (-)	
*2/*35	1 (1)	0 (-)	
*2/*41	1 (1)	3 (10)	
*3/*17	1 (1)	0 (-)	
*4/*4	2 (3)	3 (10)	
*5/*41	2 (3)	0 (-)	
*10/*41	1 (1)	0 (-)	
*35/*35	1 (1)	0 (-)	
*35/*41	0 (-)	1 (3)	
*1/*2×3^b^	3 (4)	0 (-)	
*2/*2×3^b^	2 (3)	0 (-)	
CYP2B6 Genotype^c^			0.751
*1/*1	43 (57)	18 (62)	
*1/*4	4 (5)	0 (-)	
*1/*6	23 (30)	9 (31)	
*4/*6	1 (1)	0 (-)	
*6/*6	4 (5)	1 (3)	
*CYP2C9* Genotype			0.425
*1/*1	53 (70)	19 (66)	
*1/*2	14 (18)	7 (24)	
*1/*3	6 (8)	2 (7)	
*2/*2	0 (-)	1 (3)	
*2/*3	2 (3)	0 (-)	
*3/*3	1 (1)	0 (-)	
*CYP2C19* Genotype			0.260
*1/*1	54 (71)	19 (66)	
*1/*2	22 (29)	9 (31)	
*2/*2	0 (-)	1 (3)	
*ABCB1 genotype (C3435T)*			0.266
*C/C*	24 (32)	14 (48)	
*C/T*	39 (51)	12 (41)	
*T/T*	13 (17)	3 (10)	

a Discrepancies in total numbers correspond to genotyping missing data.

b Patients with 3 functional alleles of *CYP2D6*.

c Non available data on SNP/genotype in two subjects (1 Responder and 1 Nonresponder) due to methodological problems.

**Table 3:** Phenotype frequencies of *CYP3A5, CYP2D6, CYP2B6, CYP2C9, CYP2C19 and ABCB1* between responder and nonresponder groups.

	Responders	Nonresponders	P^b^
	N = 76 (%)^a^	N = 29 (%)^a^	
*CYP3A5* Phenotype			1.000
Extensive (*1/*1 1/*3)	12 (16)	3 (10)	
Poor (*3/*3)	64 (84)	26 (90)	
*CYP2D6* Phenotype			**0.032**
Extensive (*1,*2, *3, *6, *35)	64 (84)	26 (90)	
Ultrarapid (*1xN, *2xN)	5 (7)	0 (0)	
Intermediate (*9*10,*41)	5 (7)	0 (0)	
Poor (*4/*4)	2 (3)	3 (10)	
CYP2B6 Phenotype			0,639
Extensive (*1/*1)	43 (57)	18 (62)	
Poor (*6)	27 (36)	10 (35)	
Ultrarapid (*4)	4 (5)	0 (-)	
*CYP2C9* Phenotype			0.779
Extensive (*1/*1, *1/*2)	67 (88)	26 (90)	
Intermediate (*1/*3)	6 (8)	2 (7)	
Poor (*2, *3)	3 (4)	1 (3)	
*CYP2C19* Phenotype			0.260
Extensive (*1/*1)	54 (71)	19 (66)	
Intermediate (*1/*2)	22 (29)	9 (31)	
Poor (*2/*2)	0 (-)	1 (3)	
*ABCB1* Phenotype (C3435T)			0.266
Extensive (C/C)	24 (32)	14 (48)	
Intermediate (C/T)	39 (51)	12 (41)	
Poor (T/T)	13 (17)	3 (10)	

a Discrepancies in total numbers correspond to genotyping missing data.

b Bold numbers indicate statistically significant differences between patients.

c Non available data on SNP/genotype in two subjects (1 Responder and 1 Nonresponder) due to methodological problems. One patient showed *4/*6 genotype with unknown clinical significance, therefore it was not considered in phenotype analysis.

### Methadone Dose Requirements, Plasma Concentrations, and Phenotype

We studied the mean methadone dose, *(R)-*, *(S)-* and *(R,S)-*methadone plasma concentrations by phenotype for all genes evaluated. Results for all genes studied can be found in Supplementary materials (**Table S1**). Results were essentially negative except for *CYP2D6* (see **Table 4**). We found significant differences in methadone dose requirements and plasma concentrations depending on the phenotype status in *CYP2D6*, taking patients all together: The 5 UM received higher doses of methadone compared to EM (Tukey *post-hoc* analysis) (UM 177 mg/day [SD = 96] vs. EM 95 mg/day [SD = 60], p = 0.043). PM required marginal lower doses of methadone compared to other phenotypes (87 mg/day [SD = 67]). Plasmatic concentrations showed similar results, with UM metabolizers showing higher concentrations of (R)-, (S)- and (R,S)-methadone (UM 1275 ng/ml [SD = 484] vs. EM 503 ng/ml [SD = 416], p = 0.002; UM 707 ng/ml [SD = 267] vs. EM 263 ng/ml [SD = 207], p<0.001; and UM 568 ng/ml [SD = 262] vs. EM 239 ng/ml [SD = 256], p = 0.048, respectively). A similar trend of results was observed when grouping patients as a function of clinical outcome (see **Table 4**).

**Table 4:** Mean methadone dose, *(R)*-, *(S)-* and *(R,S)-*methadone plasmatic concentrations by phenotype of *CYP2D6* in the responder and nonresponder patients.

	Metadone dose (mg/day)^a^	P^b^	*(R,S)-*Methadone (ng/ml)^c^	P^b^	*(R)-*Methadone (ng/ml)^c^	P^b^	*(S)*-Methadone (ng/ml)^c^	P^b^
	(N) mean ± SD [range]		(N) mean ± SD [range]		(N) mean ± SD [range]		(N) mean ± SD [range]	
***All patients***								
*CYP2D6* Phenotype		**0.043**		**0.002**		**<0.001**		**0.048**
Extensive	(90) 95±60 [15–400]^d^		(68) 503±416 [31–2461]^d^		(68) 263±207 [16–978]^d^		(68) 239±256 [15–1889]^d^	
Ultrarapid	(5) 177±96 [105–340]^d^		(5) 1275±484 [740–2050]^d^		(5) 707±267 [413–1084]^d^		(5) 568±262 [327–966]^d^	
Intermediate	(5) 92±60 [15–160]		(2) 368±35 [343–393]		(2) 215±30 [194–237]		(2) 152+5 [149–156]	
Poor	(5) 87±67 [30–200]		(4) 756±716 [332–1825]		(4) 416±382 [193–987]		(4) 341±336 [107–838]	
***Responder Patients***								
*CYP2D6* Phenotype		0.120		**0.002**		**<0.001**		**0.044**
Extensive	(64) 105±64 [25–400]		(56) 512±443 [31–2461]^d^		(56) 268±219 [16–978]^d^		(56) 245±276 [15–1889]^d^	
Ultrarapid	(5) 177±96 [105–340]		(5) 1275±484 [739–2050]^d^		(5) 707±267 [413–1084]^d^		(5) 568±262 [327–966]^d^	
Intermediate	(5) 92±60 [15–160]		(2) 368±35 [343–393]		(2) 216±30 [194–237]		(2) 153+5 [149–156]	
Poor	(2) 102±73 [55–200]		(2) 1164±936 [502–1825]		(2) 622±517 [256–987]		(2) 542±419 [246–838]	
***Nonresponder Patients***								
*CYP2D6* Phenotype		0.654		0.580		0.753		0.429
Extensive	(26) 72±43 [15–200]		(12) 459±264 [163–1058]		(12) 243±142 [101–577]		(12) 216±127 [62–481]	
Ultrarapid	(0) -		(0) -		(0) -		(0) -	
Intermediate	(0) -		(0) -		(0) -		(0) -	
Poor	(3) 60±33 [30–95]		(2) 349±23 [333–365]		(2) 210±23 [193–226]		(2) 139±46 [107–172]	

a Data from the 105 patients included.

b Bold numbers indicate statistically significant differences between patients.

c Plasma concentrations were obtained for 79 patients.

d Statistical significant differences were found between Ultrarapid compared to Extensive metabolizers (Tukey *post hoc* analisys) p<0.05.

Although results did not reach statistical significance, subjects homozygous carriers of the CYP2B6*6 (associated with a decreased activity of the enzyme) received lower doses of methadone (74 mg/day [SD = 24] vs. 100 mg/day [SD = 65]) and displayed higher concentrations of (*S*)-methadone plasma concentrations (347 ng/ml [SD = 279] vs. 265 ng/ml [SD = 269]) when compared with the rest of patients. (Supplementary materials,**Table S1**).

## Discussion

A number of genetic polymorphisms related to methadone metabolic disposition and transport have been examined in terms of their contribution to the clinical outcome (responders vs. nonresponders) of patients in MMT. Their contribution to clinical management and patient's satisfaction is marginal. Nevertheless, differences in methadone dosage have been found between responder and nonresponder patients. These differences cannot be attributed to genetic factors related to the pharmacokinetics of methadone but to patients' attitude in terms of accepting higher doses of methadone. Some patients (nonresponders) refuse higher doses of methadone.

Methadone patients categorized as responders and nonresponders on the basis of drug misuse while enrolled in the MMT differ on the daily dose of methadone they receive (109±68 mg/day vs. 72±43 mg/day). These differences cannot be explained by restrictions for upper limit in methadone dosage in the framework of the MMT. A potential explanation of such dosage differences and clinical outcome may come from alterations in methadone pharmacokinetics due to genetic polymorphisms regulating it.

In the context of our study, the genetic polymorphisms of *CYP3A5*, *CYP2B6*, *CYP2C9*, *CYP2C19*, and *ABCB1* examined did not influence methadone dosage. A small influence of *CYP2D6* genetic polymorphism in methadone doses and plasma concentrations was found. Mean plasma concentrations of (*R,S*)-methadone and of each enantiomer are not significantly different between responders and nonresponders, although concentrations in nonresponders were 30% lower than in responders in agreement with differences in dose requirements between both groups. Therefore, differences in clinical outcome cannot be justified on the basis of some kind of genetic differences in polymorphic drug metabolizing enzymes.

Concerning *CYP2D6* genetic polymorphism, its contribution on methadone metabolic disposition and dosage is controversial. Several reports suggest that its contribution is negligible [17], [58], while others have shown that specific inhibitors of CYP2D6 as paroxetine, markedly influence methadone disposition [29], [30]. Five CYP2D6 UM subjects were identified among responder patients while none among nonresponders. The UM phenotype has been associated to lack of satisfaction of methadone treatment [6] and with lower trough *(R,S)*-methadone plasma levels compared to other CYP2D6 phenotypes [14], suggesting an increased methadone metabolic disposition. In this study, UM patients required high doses of methadone (about 180 mg), about twice to those provided to EM patients. Nevertheless this increased request of methadone is not related with an increased metabolic disposition, as plasma concentrations of methadone and its enantiomers are the highest among all *CYP2D6* phenotypes. The five PM patients included in this study (3 in the nonresponders and 2 in the responders groups) required marginally lower methadone doses and display twice methadone plasma concentrations of EM subjects, being methadone dosage quite similar. Observations made in UM and PM patients are contradictory in terms of methadone plasma concentrations (but not in terms of dosage) and tune down the relevance of CYP2D6 in methadone metabolism. Discrepancies between *CYP2D6* genotype and phenotype in terms of methadone metabolism have been already described [34]. The observed discrepancies could be related to interactions with other drugs as CYP2D6 has been implicated in the metabolism of other medications [59]. The effect of drug interactions could not be discarded in our results as a high proportion of patients were taking concomitant medications (65%). Nevertheless present observations should apply to EM patients, but not to PM patients (homozygous for non-functional allelic variants) with a non-functional enzyme or to UM (homozygous for more than two functional allelic variants) the most susceptible to drug interactions but also, those requiring the larger doses. A recent report suggests that CYP2D6, a non-inducible hepatic enzyme, may be induced at the brain level by nicotine [60]. As almost all participants were smokers, there may be dissociation between plasma concentrations, hepatic metabolism and genotype, and brain drug requirements. According to previous publications [61] there is a pharmacodynamic interaction between methadone treatment and cigarette smoking; methadone and nicotine (smoking) share some effects: increase the ratings of euphoria and ameliorate negative mood. The contribution of CYP2D6 to methadone metabolism as well the interaction with smoking deserves further studies.

Regarding the results of CYP2B6, although non-statistically significant, methadone doses and methadone plasma concentrations are in agreement with previous research reports: patients homozygous for the *6 allele, received lower doses of methadone and those patients showed higher plasma concentrations of *(S)-*methadone, confirming previous findings [14]. Increased *(S)-*methadone plasma concentrations are related with an enantioselective methadone metabolic disposition towards the inactive (S)-enantiomer regulated by CYP2B6*6 [19]. Also, in a recent report examining methadone concentrations in post-mortem blood in methadone-related deaths, it was concluded that the risk of methadone fatality may be related in part with the CYP2B6*6 allele [62]. When we look at responder status, 4 out of 5 slow metabolizers were classified as responders to methadone treatment.

It is apparent from the present study that interindividual pharmacokinetic differences among patients can be compensated by clinical management of the doses of methadone (e.g. dose requirements of UM patients). Although the absence of restrictions in methadone dosage in our MMT program, the clinical impression is that some patients with poor response to MMT do not accept increases in their methadone dose [9]. It could be hypothesized that those patients show significant adverse events associated to methadone. *(S)*-methadone has been previously associated to adverse responses of (*R,S*)-methadone as negative mood effects –tension, fatigue, confusion…-.[5], [63] No significant differences in the *(S)*-methadone plasma concentrations have been detected, nor in the *(R)/(S)* ratio in this sample. Other possible explanations could be a pharmacodynamic influence in the reluctance of a considerable group of patients to increase methadone dose. The candidate gene *OPRM1* has previously been related to opioid treatment response, mainly in analgesia and alcohol dependence [47], [64], [65]. The more commonly studied SNP (A118G), in the mu-opioid receptor gene can affect opioid function. Carriers of the homozygous variant (GG) require higher opiate doses to achieve pain relief when they are treated with morphine [66]. However, when genetic variability of this receptor has been considered in MMT results have been negative [67] as in our study. These negative findings may suggest that these variants are specifically involved in the heroin dependence phenotype but not in the individual differences in the response to methadone treatment in heroin addiction.

An influence of a on the *DRD2* gene promoter (rs1800497 C>T) has been associated with both, the risk of opiate addiction, leading to the necessity of methadone substitution therapy, and the course of this therapy in terms of dosage requirements [68]. Other pharmacodynamic influences in those patients could be a difference related to the activation of kappa opioid receptors. Kappa opioid receptors have been involved in the response to drugs (cocaine, alcohol and opiates) [69] in opiate withdrawal and stress responsivity [70]; kappa agonists lower the levels of dopamine in the nucleus accumbens and act in a counter-modulatory manner to attenuate the increase in dopamine levels [71] and induce a negative mood state [70].

The negative results in terms of the contribution of genotypes and phenotypes of drug metabolizing enzymes examined in this study on the clinical outcomes of MMT are consistent with previous data [14], [37], [72]. Classically, treatment response has been evaluated in terms of retention in treatment and opioid consumption measured by urine drug tests. In recent years, aspects as patients' satisfaction with the MMT program are considered important in the outcome [73], [74] also, personal attitudes as coping self-efficacy have recently received attention [4].

Among other factors to take into consideration in response to methadone maintenance treatment is the duration of treatment. As seen in our results, responder patients stayed in treatment more than twice than nonresponders (52 *vs* 21 months). Some studies have shown that the results obtained after treatment over a period of less than 3 months were comparable to those obtained after no treatment at all [75] and others described reduction in drug use when patients remained in treatment for at least one year [76].

The present findings should be interpreted taking into account some limitations of the study. Firstly, the sample size was small; complex study procedures in the framework of a longitudinal design (urine testing, blood analyses for genotyping, time consuming interviews) can result in a non-negligible number of patients with incomplete follow-up data. It is also remarkable that patients with poorer outcomes (for example, more illicit drug use) were more reluctant to accept to participate in clinical studies; this could imply a bias in the study results, but, on the other hand, to offer a payment for the participation it is not acceptable on ethical grounds. Lastly, we cannot exclude a risk of stratification effect, although all subjects were Caucasian.

Globally from the present study, it is apparent that interindividual pharmacokinetic differences among patients can be compensated by clinical management of the doses of methadone, with little influence, if any, from pharmacogenetics of drug metabolizing enzymes and protein transporters. The interest should to be driven towards the genetics of pharmacodynamics in methadone treatment response.

## Supporting Information

Table S1 Mean methadone dose requirements of patients (n = 105) and *(R)*-, *(S)-* and *(R,S)-*methadone plasma concentrations (n = 79) according phenotypes of genes evaluated (*CYP3A5, CYP2D6, CYP2B6, CYP2C9, CYP2C19 and ABCB1*).
(DOC)
