# Supplementary material for: Contribution of Cytochrome P450 and ABCB1 Genetic Variability on Methadone Pharmacokinetics, Dose Requirements, and Response
Source: PLoS One. 2011 May 12;6(5):e19527. doi: 10.1371/journal.pone.0019527 (PMC3093392; doi:10.1371/journal.pone.0019527)
Supplement: Table S1 — Mean methadone dose requirements of patients (n = 105) and (R)-, (S)- and (R,S)-methadone plasma concentrations (n = 79) according phenotypes of genes evaluated (CYP3A5, CYP2D6, CYP2B6, CYP2C9, CYP2C19 and ABCB1). (DOC) [file pone.0019527.s001.doc]

**Table S1**. Mean methadone dose requirements of patients (n=105) and *(R)*-, *(S)-* and *(R,S)-*methadone plasma concentrations (n=79) according phenotypes of genes evaluated (*CYP3A5*, *CYP2D6,*  *CYP2B6, CYP2C9, CYP2C19 and ABCB1)* ^a^

|  | **Metadone dose (mg/day)^b^** | **P^c^** | ***(R,S)-*Methadone (ng/ml)^d^** | **P^c^** | ***(R)-*Methadone (ng/ml)^d^** | **P^c^** | ***(S)*-Methadone (ng/ml)^d^** | **P^c^** |
| --- | --- | --- | --- | --- | --- | --- | --- | --- |
|  | **(N) mean + SD [range]** |  | **(N) mean + SD [range]** |  | **(N) mean + SD[range]** |  | **(N) mean + SD [range]** |  |
| *CYP3A5* Phenotype |  | 0.312 |  | 0.540 |  | 0.516 |  | 0.657 |
| Extensive | (2) 30 + 7 [25-35] |  | (1) 40- |  | (1) 20- |  | (1) 20- |  |
| Poor | (13) 100 + 56 [35-200] |  | (12) 568 + 574 [40-1825] |  | (12) 310 + 329 [20-987] |  | (12) 258+ 251 [20-838] |  |
| Very Poor | (90) 100 + 65 [15-400] |  | (66) 568 + 450 [31-2461] |  | (66) 300 + 226 [16-1084] |  | (66) 268 + 271 [15-1889] |  |
| *CYP2D6* Phenotype |  | **0.043** |  | **0.002** |  | **<0.001** |  | **0.048** |
| Extensive | (90) 95 + 60 [15-400] ^e^ |  | (68) 503 + 416 [31-2461] ^e^ |  | (68) 263 + 207 [16-978] ^e^ |  | (68) 239 + 256 [15-1889] ^e^ |  |
| Ultrarapid | (5) 177 + 96 [105-340]^e^ |  | (5) 1275 + 484 [740-2050] ^e^ |  | (5) 707 + 267 [413-1084] ^e^ |  | (5) 568 + 262 [327-966] ^e^ |  |
| Intermediate | (5) 92 + 60 [15-160] |  | (2) 368 + 35 [343-393] |  | (2) 215 + 30 [194-237] |  | (2) 152 + 5 [149-156] |  |
| Poor | (5) 87 + 67 [30-200] |  | (4) 756 + 716 [332-1825] |  | (4) 416 + 382 [193-987] |  | (4) 341 + 336 [107-838] |  |
| *CYP2B6* Phenotype |  | 0.372 |  | 0.806 |  | 0.927 |  | 0.608 |
| Extensive | (98) 100 + 65 [15-400] |  | (74) 569 + 471 [31-2461] |  | (74) 304 + 245 [16-1084] |  | (74) 265 + 269 [15-1889] |  |
| Poor^f^ | (5) 74 + 24 [35-95] |  | (3) 637 + 508 [91-1097] |  | (3) 290 + 229 [45-499] |  | (3) 347 + 279 [46-598] |  |
| *CYP2C9* Phenotype |  | 0.483 |  | 0.158 |  | 0.091 |  | 0.361 |
| Extensive | (72) 103 + 71 [15-400] |  | (54) 628 + 510 [40-2461] |  | (54) 338 + 260 [20-1084] |  | (54) 290 + 301 [20-1889] |  |
| Intermediate | (21) 90 + 45 [20-190] |  | (17) 445 + 340 [31-1178] |  | (17) 217 + 175 [16-684] |  | (17) 228 + 176 [15-603] |  |
| Poor | (12) 83 + 45 [25-190] |  | (8) 356 + 297 [40-929] |  | (8) 196 + 187 [20-567] |  | (8) 159 + 113 [20-362] |  |
| *CYP2C19* Phenotype |  | 0.420 |  | 0.578 |  | 0.999 |  | 0.328 |
| Extensive | (73) 101 + 69 [15-400] |  | (54) 581 + 497 [40-2461] |  | (54) 298 + 241 [20-1084] |  | (54) 283 + 303 [20-1889] |  |
| Intermediate | (31) 95 + 50 [15-190] |  | (25) 518 + 403 [31-1607] |  | (25) 298 + 251 [16-978] |  | (25) 220 + 161 [15-629] |  |
| Poor | (1) 20 – |  | (0) - |  | (0) - |  | (0) - |  |
| *ABCB1* Phenotype |  | 0.828 |  | 0.352 |  | 0.775 |  | 0.163 |
| Extensive | (38) 97 + 61 [15-270] |  | (25) 667 + 621 [40-2461] |  | (25) 326 + 273 [20-1084] |  | (25) 341 + 396 [20-1889] |  |
| Intermediate | (51) 102 + 69 [25-400] |  | (41) 494 + 341 [31-1282] |  | (41) 282 + 220 [16-929] |  | (41) 212 + 150 [15-598] |  |
| Poor | (16) 91 + 56 [15-190] |  | (13) 568 + 474 [40-1607] |  | (13) 293 + 261 [20-978] |  | (13) 275 + 228 [20-697] |  |

^a^ Discrepancies in total numbers correspond to genotyping missing data

^b^ Data on methadone dose was obtained in 105 patients

^c^ Bold numbers indicate statistically significant differences between patients

^d^ Plasma concentrations were obtained in 79 patients

^e^ Statistical significant differences were found between Ultrarapid compared to Extensive metabolizers (Tukey *post hoc* analisys) p< 0.05

^f^ Homozygous carriers of *CYP2B6*6*
